# Cuproptosis scoring system to predict the clinical outcome and immune response in bladder cancer

**DOI:** 10.3389/fimmu.2022.958368

**Published:** 2022-08-04

**Authors:** Qiang Song, Rui Zhou, Fangpeng Shu, Wen Fu

**Affiliations:** ^1^ Department of Urology, Guangzhou Women and Children’s Medical Center, National Children’s Medical Center for South Central Region, Guangzhou Medical University, Guangzhou, China; ^2^ Department of Urology, The First Affiliated Hospital of Nanjing Medical University, Nanjing, China

**Keywords:** bladder cancer, cuproptosis, prognosis, tumor microenvironment, immunotherapy

## Abstract

Cuproptosis is a novel copper ion-dependent cell death type being regulated in cells, and this is quite different from the common cell death patterns such as apoptosis, pyroptosis, necroptosis, and ferroptosis. Interestingly, like with death patterns, cuproptosis-related genes have recently been reported to regulate the occurrence and progression of various tumors. However, in bladder cancer, the link between cuproptosis and clinical outcome, tumor microenvironment (TME) modification, and immunotherapy is unknown. To determine the role of cuprotosis in the tumor microenvironment, we systematically examined the characteristic patterns of 10 cuproptosis-related genes in bladder cancer (BLCA). By analyzing principal component data, we established a cuproptosis score to determine the degree of cuproptosis among patients. Finally, we evaluated the potential of these values in predicting BLCA prognosis and treatment responses. A comprehensive study of the mutations of cuproptosis-related genes in BLCA specimens was conducted at the genetic level, and their expression and survival patterns were evaluated using The Cancer Genome Atlas (TCGA) and Gene Expression Omnibus (GEO). Two cuproptosis patterns were constructed based on the transcription level of 10 cuproptosis-related genes, featuring differences in the prognosis and the infiltrating landscape of immune cells (especially T and dendritic cells) with interactions between cuproptosis and the TME. Our study further demonstrated that cuproptosis score may predict prognosis, immunophenotype sensitivity to chemotherapy, and immunotherapy response among bladder cancer patients. The development and progression of bladder cancer are likely to be influenced by cuproptosis, which may involve a diverse and complex TME. The cuproptosis pattern evaluated in our study may enhance understanding of immune infiltrations and guide more potent immunotherapy interventions.

## Introduction

Globally, bladder cancer is one of the top 10 most common types of cancer, with 550,000 new cases and 200,000 deaths annually (1). The World Health Organization (WHO) categorizes it based on its standardized histomorphological features into high-grade and low-grade cancers. Researchers from several independent groups have reported genes and mutations associated with low-grade non-muscle-invasive bladder cancer (NMIBC; PIK3CA, FGFR3, STAG2, and RTK/RAS/RAF pathway genes) and high-grade muscle-invasive bladder cancer (MIBC; p53, RB1, MDM2, ERBB2, CDKN2A, ARID1A, and KDM6A), which are relevant to prognosis, prediction, and treatment of bladder cancer. In particular, the CDKN2A mutation has been identified as a high-risk factor for MIBC (2, 3). Platinum-based chemotherapy was the mainstay of systematic management of MIBC. Recent years have seen the emergence of new treatment options such as PD-1/PD-L1 inhibitors, but immunotherapy is still in its infancy, and more efforts are urgently needed (4). Thus, developing new ideas for the development of immunotherapy for bladder cancer is of immense clinical significance.

Research in the field of life science has always been focused on the phenomenon of cell death, which is a normal part of living. Cell death patterns differ based on the mechanism of occurrence, including apoptosis, ferroptosis, pyroptosis, and necroptosis. Researchers have recently focused their attention on ferroptosis, a classic cell death mechanism described in 2012 (5). These regulated cell death (RCD) subroutines differ in the initiating stimuli, intermediate activation events, and terminal effects. Heavy metal ions are essential micronutrients in the body, but too little or too much of the metal can lead to cell death (6). Copper, like ferric ions, is an essential trace element in all life, usually present in extremely low concentrations in mammalian cells. If the copper ion concentration in the cell exceeds the threshold required to maintain homeostasis, it will also cause cytotoxicity (7). During research on copper ion-carrying cell death, Todd Golub’s team discovered a new pattern of cell death involving copper ions in cells that is dependent on and regulated by copper ions termed cuproptosis (8). The mechanism of cuproptosis involves the binding of copper ions directly to the lipoylated components of the tricarboxylic acid (TCA) cycle, which lead to abnormal aggregation of fatty acylated proteins as well as loss of iron–sulfur cluster protein ultimately resulting in proteotoxic stress response-mediated cell death. Further, this study identified seven genes (FDX1, LIAS, LIPT1, DLD, DLAT, PDHA1, and PDHB) that promote cuproptosis, as well as three genes (MTF1, GLS, and CDKN2A) that inhibit it. In previous studies, it was found that the copper depletion caused by tetrathiomolybdate (TM) influences immune response, and intratumoral copper ions may be able to regulate PD-L1 expression and influence tumor immune escape (9, 10). Consistently, the latest report of Bian et al. also showed that cuproptosis was significantly correlated with immune cell infiltration and PD-1 expression in clear cell renal cell carcinoma (ccRCC) (11). In addition, copper, platinum, and iron as raw materials have been extensively studied in the development of drug delivery systems, showing promising prospects for a range of anticancer purposes (12). Many potential mechanisms for utilizing the biological functions of copper ions to treat cancer are also being investigated (13). However, the role of cuproptosis in the immune microenvironment and immunotherapy of bladder cancer remains uncertain.

As a part of our study, we constructed two cuproptosis patterns, each associated with a different prognosis and tumor microenvironment (TME) characteristic. We are the first to propose the use of the cuproptosis score to quantify the cuproptosis pattern of each bladder cancer (BLCA) patient based on the expression profile of cuproptosis-related genes (CUGs). This scoring mechanism can help doctors in developing more effective and personalized immunotherapy strategies.

## Materials and methods

### Data collection

The mRNA expression profiles, copy number variation (CNV) files, somatic mutation data, pathology slides, and corresponding clinical information on bladder cancer were retrieved from The Cancer Genome Atlas (TCGA)-BLCA (https://portal.gdc.cancer.gov/) program, and GSE16671 and GSE13507 in Gene Expression Omnibus (GEO) (https://www.ncbi.nlm.nih.gov/geo/) were used to determine the clinical characteristics and gene expression data. Among these, patients lacking important clinical or survival parameters are eliminated from further analysis. The ‘limma 3.52.2’ package was used to post-process and normalize the raw reads from the above data by R 4.1.1 software. The pathology image data (formalin-fixed paraffin-embedded (FFPE) slide) were downloaded from the Genomic Data Commons (GDC; https://portal.gdc.cancer.gov/).

### Consensus clustering analysis of cuproptosis-related genes

Based on the expression profiles of 10 CUGs, unsupervised classes of TCGA-BLCA datasets are estimated with the consensus clustering method using the ‘ConsensusCluster-Plus 1.60.0’ package in R (14), and two clusters are obtained. t-Distributed stochastic neighbor embedding (t-SNE) was used to verify the clusters based on the expression profiles of the above genes. A gene set variation analysis (GSVA) was performed using the Kyoto Encyclopedia of Genes and Genomes (KEGG) gene set (C2.cp.kegg.V7.4) to identify differences in biological function between the two clusters (15).

### Relationship between cuproptosis patterns with the clinical characteristics and prognosis of bladder cancer

To evaluate the clinical significance of the two clusters identified by consensus clustering, we compared the associations between cuproptosis patterns, clinicopathological features, and survival outcomes. The characteristics of the patients included gender, age, T-stage, grade, and survival status. Furthermore, the differences in overall survival (OS) among different clusters were evaluated through the Kaplan–Meier analysis generated by the ‘Survival 3.3-1’ and ‘Survminer 0.4.9’ packages of R.

### Associations of gene clusters with tumor microenvironment and human leukocyte antigen in bladder cancer

To evaluate the immune and stromal scores of each BLCA patient, we used the ESTIMATE algorithm (16). In addition, the CIBERSORT algorithm (17) was applied to determine the proportions of immune cell subsets in each BLCA sample. Additionally, the levels of human leukocyte antigen (HLA), immune cell infiltration, and the immune cell functions in the BLCA among different patterns were also calculated with the single-sample gene set enrichment analysis (ssGSEA) algorithm (18). Through TCGA Pathology Slides, we were able to confirm the above analysis.

### Differentially expressed genes and cuproptosis score

Differentially expressed genes (DEGs) in the distinct cuproptosis subgroups were identified using the ‘limma 3.52.2’ package of R with criteria of |log2-fold change (FC)| ≥ 1 and *p*-value < 0.05. Gene Ontology (GO) and KEGG analyses were used to identify related gene functions and enriched pathways using the ‘clusterProfiler 1.4.0’ package in R. To quantify the cuproptosis patterns of individual BLCA patients, cuproptosis scores were calculated. The Boruta algorithm was performed to reduce the size of the cuproptosis gene signatures A and B, and principal component analysis was used to extract principal component 1 as signature score (19). Then the score of each patient is calculated by a method, as follows: cuproptosis score = ∑PC1A − ∑PC1B, where PC1A means the first component of signature A, and PC1B represents the first component of signature B. By using the Kruskal–Wallis test, cuproptosis scores of molecular patterns or gene clusters were evaluated.

### Clinical significance and stratification analyses of the prognostic cuproptosis score

We performed univariate and multivariate Cox proportional hazards regression to determine whether cuproptosis score is an independent predictor of prognosis. Based on multiple clinical variables, we then carried out a classification analysis to determine whether the cuproptosis score still had predictive value in different subgroups. To assess the clinical accuracy of cuproptosis score, nomogram, calibration plots, and decision curve analysis (DCA) was conducted. Additionally, gene set enrichment analysis (GSEA) enrichment analysis was performed between high and low cuproptosis scores. The criteria of |log2-fold change (FC)| ≥ 1 and *p*-value < 0.05 were considered significantly enriched. In addition, we used Wilcoxon rank-sum test to compare the differences in immune cell infiltration, immune cell functions, and expression of 10 CUGs between the high and low cuproptosis score groups. Finally, TCGA Pathology Slides confirmed the above analysis.

### Assessment of mutation and drug sensitivity

The association between cuproptosis score and tumor mutational burden (TMB) score was examined. In addition, data on somatic mutations using a mutation annotation format (MAF) file were visualized using the R package ‘maftools 2.12.0’. The Genomics of Drug Sensitivity in Cancer (GDSC; https://www.cancerrxgene.org/) database was utilized to evaluate the sensitivity of each BLCA patient to several chemotherapy drugs, and IC50 was quantified *via* the ‘pRRophetic’ package of R. Next, we predicted the response to immune checkpoint blockade (ICB) through the TIDE website (http://tide.dfci.harvard.edu/login/). With the use of the Wilcoxon test, the difference in TIDE score between high and low cuproptosis groups was compared. Additionally, both groups were predicted to respond to anti-PD1 or anti-CTLA4.

### Screening of small molecular drugs

DEGs between the high and low cuproptosis score groups were identified using the ‘limma 3.52.2’ package using the criteria of |log2-fold change (FC)| ≥ 1 and *p*-value < 0.05. GO and KEGG enrichment analyses were conducted by the ‘clusterProfiler 1.4.0’ R package. As a final step, the upregulated and downregulated genes were uploaded to the CMap database, and candidate small-molecule drugs and their mechanisms of action were examined using the CMap mode of action (MoA).

### Statistical analysis

All statistical analyses were performed in R software (version 4.1.1) and Perl, and a *p* < 0.05 value was considered statistically significant.

## Results

### The genetic characteristics and transcriptional alterations of cuproptosis-related genes in bladder cancer

**Figure 1:**
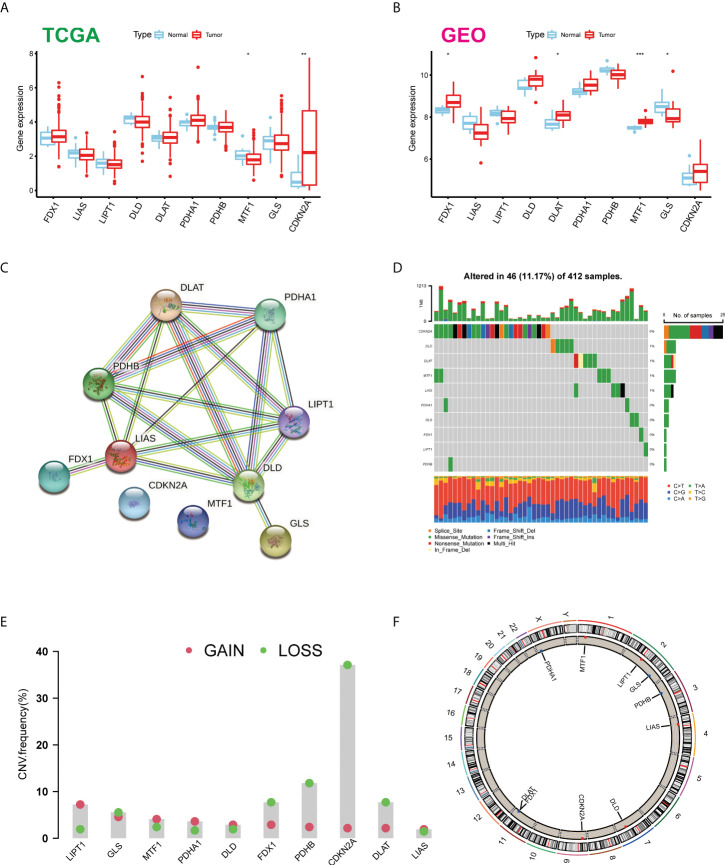
Expression distributions and genetic mutational landscape of CUGs in BLCA. Expression distributions of 10 CUGs between BLCA and normal tissues in the **(A)** TCGA-BLCA and **(B)** GSE166716 datasets. **(C)** The PPI network acquired from the STRING database among the CUGs. **(D)** Genetic alteration on a query of CUGs. **(E)** Frequencies of CNV gain, loss, and non-CNV among CUGs. **(F)** Circus plots of chromosome distributions of CUGs (*p < 0.05; **p < 0.01; ***p < 0.001). CUGs, cuproptosis-related genes; BLCA, bladder cancer; TCGA, The Cancer Genome Atlas; PPI, protein–protein interaction.

Firstly, we determined the expression levels of 10 CUGs in tumor and normal specimens using TCGA-BLCA and GSE166716 datasets. There was a total of six DEGs identified (**Figures 1A, B**). Through the protein–protein interaction (PPI) analysis of the String website, we found that LIAS and DLD are the central genes of the network (**Figure 1C**). In addition, we calculated the incidence of CNVs and somatic mutations in 10 CUGs in BLCA. Based on **Figure 1D**, 46 (11.17%) of the 412 BLCA samples were mutated, and the results showed that the CDKN2A mutation rate was the highest among the 10 CUGs, which was consistent with previous studies (3). Next, we investigated the copy number alterations of these 10 CUGs and found that copy number alterations were common among them. Among them, the CNV of LPT1 increased, while FDX1, PDHB, CDKN2A, and DLAT had an extensive decrease in CNV (**Figure 1E**). **Figure 1F** displays the location of CNV of these CUGs on chromosomes (*p*-value < 0.05 was considered statistically significant).

### Identification of cuproptosis subtypes in bladder cancer

**Figure 2:**
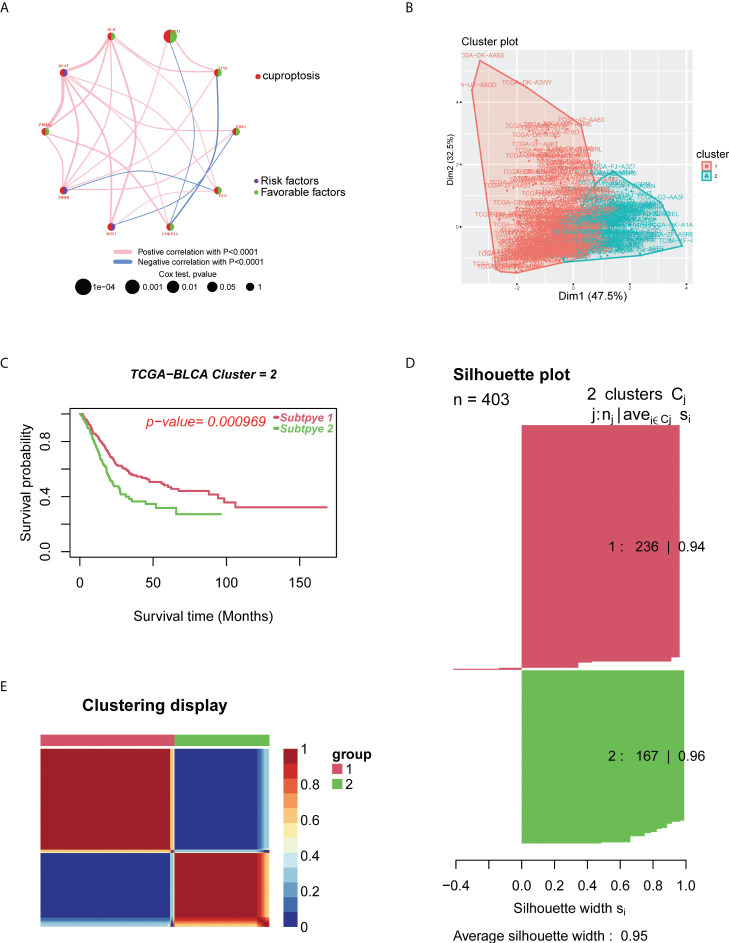
Identification of cuproptosis subtypes in BLCA. **(A)** A network of correlations including CUGs in TCGA cohort. **(B)** t-SNE of the mRNA expression profiles of CUGs from the BLCA samples in TCGA dataset confirmed the two clusters: C1 and C2. **(C)** Kaplan–Meier curves for the two molecular patterns of BLCA patients. **(D)** Silhouette plot of the two clusters. **(E)** Clustering display of the molecular subgroups. BLCA, bladder cancer; CUGs, cuproptosis-related genes; TCGA, The Cancer Genome Atlas; t-SNE, t-distributed stochastic neighbor embedding.

To explore the relationship between cuproptosis and tumorigenesis, 403 patients from TCGA-BLCA and 165 patients from GSE13507 of BLCA were included in this study. In **Figure S1** and **Table S1**, we present the prognostic value of 10 CUGs for patients with BLCA analyzed by UniCox. Then, the network of CUG interactions, regulatory relationships, and their survival significance in BLCA patients is shown in **Figure 2A**. We then conducted unsupervised non-negative matrix factorization (NMF) clustering of TCGA-BLCA samples based on the mRNA expression level of 10 CUGs. Molecular clustering effects and cophenetic correlation analysis are used to comprehensively determine the optimal *k*. We tried *k* from 2 to 5; among all *k* values, *k* = 2 produced the best results in terms of clustering, OS analysis, and contours (**Figures 2B-E**). The two subgroups were designated as C1 and C2 (*p*-value < 0.05 was considered statistically significant).

### Different clinical and tumor microenvironment features of the two clusters in bladder cancer

**Figure 3:**
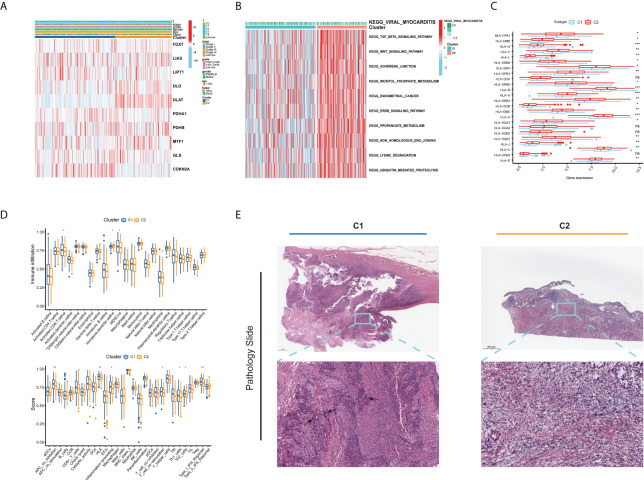
Different clinical and TME features of the two cuproptosis subtypes in BLCA. **(A)** Heatmap depicts the correlation between the subtypes and different clinicopathological characteristics. **(B)** GSVA enrichment analysis of biological pathways between the two distinct subtypes. **(C)** Boxplots show variations in mRNA expression of HLAs in the two cuproptosis subtypes. **(D)** Boxplots show abundance of 23 infiltrating immune cell types and differences in immune scores in the two cuproptosis subtypes. **(E)** Representative pictures of pathological H&E staining of two cuproptosis subtypes (*p < 0.05; **p < 0.01; ***p < 0.001; Ns, not significant). TME, tumor microenvironment; BLCA, bladder cancer; GSVA, gene set variation analysis; HLAs, human leukocyte antigens.

As displayed in **Figure 3A**, the gene expression and clinical variables of the two clusters were compared, and significant differences in the expression of CUGs and clinical characteristics were identified. As a result of GSVA, Cluster 2 showed significant enrichment in tumorigenesis-related pathways, such as the TGF-β signaling pathway, WNT signaling pathway, adherens junction pathway, and ERBB signaling pathway (**Figure 3B**). Additionally, we investigated the impact of cuproptosis on TME. The results show that most HLAs are highly expressed in Cluster 2 (**Figure 3C**). In contrast, more lymphocyte infiltration and higher immune score were observed in C1. It is hypothesized that this is caused by negative immune feedback during the antitumor process (**Figure 3D**). TCGA Pathology Slides confirmed that immune cell infiltration was greater in the tumor nests of Cluster 1 patients than in Cluster 2 patients (**Figure 3E**) (*p*-value < 0.05 was considered statistically significant).

### Prognosis and tumor microenvironment features in four cuproptosis gene clusters for bladder cancer

**Figure 4:**
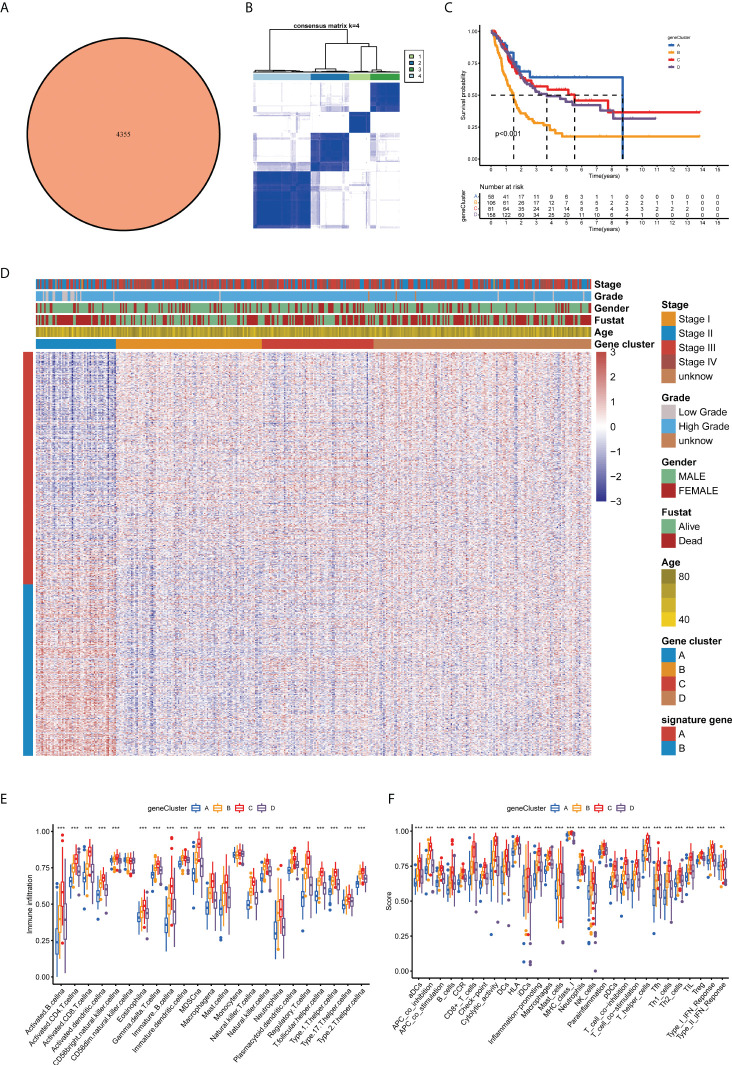
Prognosis and TME characteristics in four cuproptosis gene clusters for BLCA patients. **(A)** Differential expression analysis between the two cuproptosis subtypes: 4,355 common DEGs. **(B)** Consensus matrix heatmap defining four gene clusters according to the prognostic DEGs. **(C)** Kaplan–Meier survival analysis for patients in the four gene clusters. **(D)** Clinical features of the four cuproptosis gene clusters. Boxplots show **(E)** abundance of 23 infiltrating immune cell types and **(F)** differences in immune scores in the four gene clusters (**p < 0.01; ***p < 0.001). TME, tumor microenvironment; BLCA, bladder cancer; DEGs, differentially expressed genes.

Furthermore, we investigated the biological activities of the two cuproptosis subgroups. Based on differential expression analysis using the ‘limma’ package between the two clusters, we identified 4,355 common DEGs (**Figure 4A**). Functional enrichment analysis was then performed based on these DEGs. The DEGs associated with these cuproptosis subgroups are mainly involved in biological processes related to RNA splicing (**Figure S2A**). Based on the KEGG analysis, immune-related pathways are enriched (**Figure S2B**), indicating that cuproptosis is involved in the immune regulation of BLCA. Following this, we performed a uniCox analysis to estimate the survival significance of these 4,355 DEGs, and 770 genes were screened out with a criterion of *p* < 0.05 for subsequent analysis **(** **Table S2** **)**. As a next step, the unsupervised clustering method was proposed to divide TCGA-BLCA cohort into four gene clusters according to these prognostic genes **(**A–D; **Figures 4B**, **S3A, B**). The Kaplan–Meier curves showed that BLCA patients with gene cluster B had the shortest OS time, whereas patients in gene cluster A possessed a pessimistic prognosis **(** *p* < 0.05; **Figure 4C** **)**. Based on the cuproptosis gene signatures A and B, the dimension reduction was presented by the Boruta algorithm. In the heatmap transcriptomic profile, the 770 most abundant DEGs were displayed (**Figure 4D**). The cuproptosis gene clusters showed a significant difference in the expression of CUGs, as expected by the cuproptosis subgroup (**Figure S3C**). As a result, clusters B and C had higher immune cell infiltration and immune function scores than clusters A and D (**Figures 4E,F**). Based on the consistency of the prognosis and TME features of the four gene clusters, this classification can be considered reasonable and credible (*p*-value < 0.05 was considered statistically significant).

### Development and validation of the cuproptosis scoring system for bladder cancer

**Figure 5:**
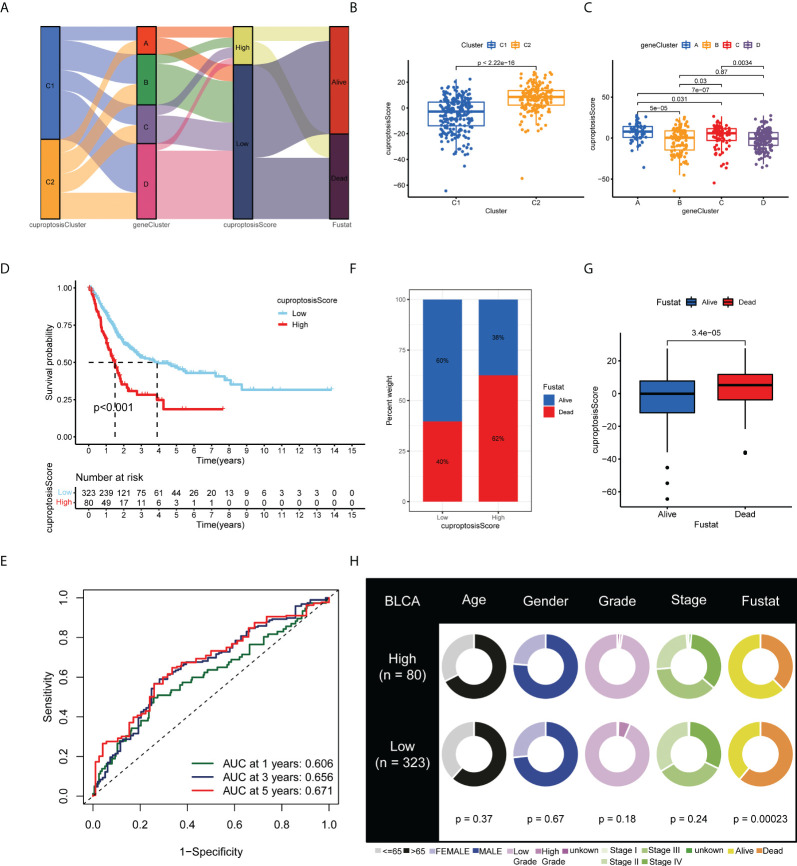
Development and validation of the cuproptosis scoring system for BLCA. **(A)** Alluvial diagram of two cuproptosis subtypes, four gene clusters, cuproptosis scores, and clinical outcomes. Differences in cuproptosis score between **(B)** the two cuproptosis subtypes and **(C)** the four gene clusters. **(D)** Kaplan–Meier analysis of the OS between the two cuproptosis score groups. **(E)** ROC curves to predict the sensitivity and specificity of 1-, 3-, and 5-year survival according to the cuproptosis score. **(F)** Histogram and **(G)** boxplot depict the distribution of survival status of BLCA patients in the two groups. **(H)** Clinical characteristics for the high and low cuproptosis score groups. BLCA, bladder cancer; OS, overall survival; ROC, receiver operating characteristic.

Based on the 770 prognostic DEGs, we calculated the cuproptosis score for each BLCA sample using principal component analysis (PCA). A cuproptosis score of high or low was assigned to all patients. **Figure 5A** illustrates the distribution of cuproptosis scores within the two clusters of cuproptosis, the three gene clusters, and the patient’s survival status. Moreover, we observed a significant difference between the cuproptosis clusters and gene clusters in the cuproptosis score (**Figures 5B, C**). Compared with patients with low cuproptosis scores, those with high cuproptosis scores usually indicate a poor prognosis (*p* < 0.001; **Figure 5D**), and the AUC values of 1-year, 3-year, and 5-year OS were 0.606, 0.656, and 0.671, respectively (**Figure 5E**). The histogram and boxplots reveal that patients with high scores have significantly higher mortality rates than patients with low scores (*p* < 0.001; **Figures 5F, G**). As a result of our findings, the cuproptosis score correlated significantly with survival status in TCGA-BLCA cohort, but not with other clinical factors, such as age, gender, grade, and stage (**Figure 5H**) (*p*-value < 0.05 was considered statistically significant).

### Nomogram construction for predicting the prognosis of patients

**Figure 6:**
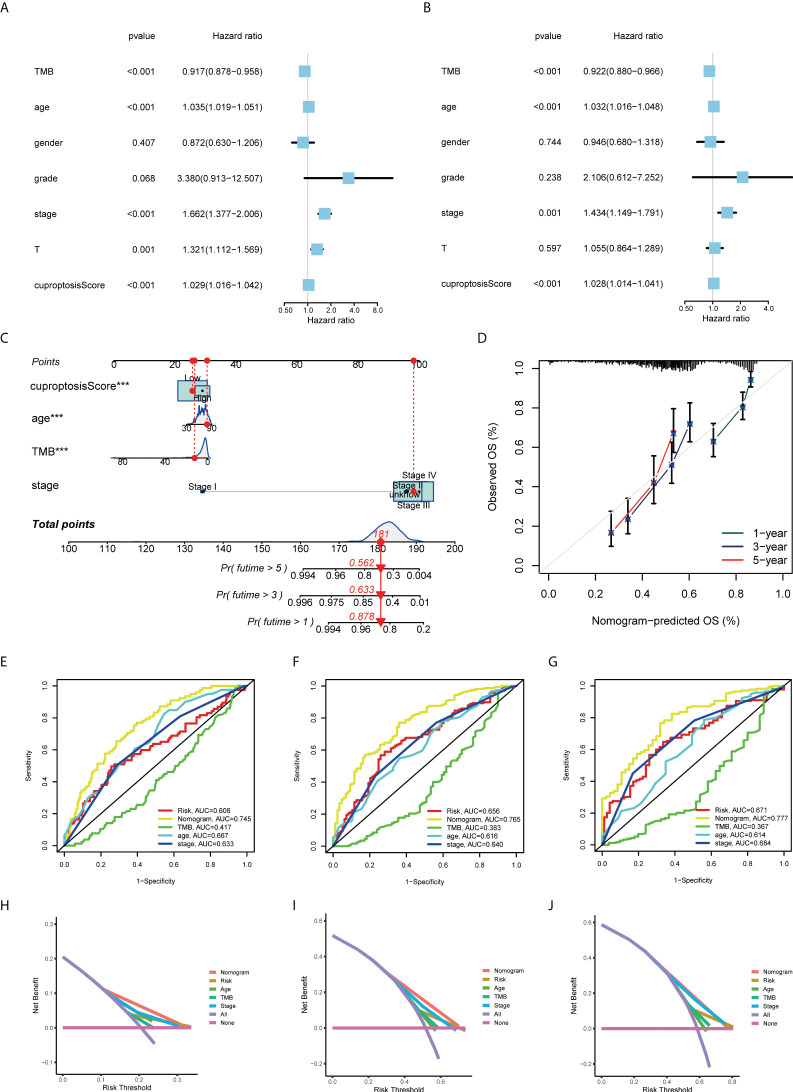
Construction and validation of a nomogram for predicting the prognosis of BLCA patients. The **(A)** uniCox and **(B)** multiCox analyses explored the independent prognostic value of cuproptosis score and multiple clinical factors. **(C)** Nomogram for predicting the 1-, 3-, and 5-year OS of BLCA patients in TCGA-BLCA cohort. **(D)** Calibration curves for validating the established nomogram. **(E–G)** The ROC curves of the nomograms compared for 1-, 3-, and 5-year OS in BLCA patients, respectively. **(H–J)** The DCA curves of the nomograms compared for 1-, 3-, and 5-year OS in BLCA patients, respectively (***p < 0.001). BLCA, bladder cancer; OS, overall survival; TCGA, The Cancer Genome Atlas; ROC, receiver operating characteristic; DCA, decision curve analysis.

Based on uniCox and multiCox analyses, we analyzed the independent prognostic value of cuproptosis score for BLCA patients, as well as the prognostic value of multiple clinical factors (**Figures 6A, B**; **Tables 1**, **S3**). Using the cuproptosis score and clinical parameters, we constructed a nomogram to estimate 1-, 3-, and 5-year OS for patients with BLCA (**Figure 6C**). The calibration curves of this nomogram showed high consistency between the observed and predicted values **Figure 6D**). In the AUC experimental results of other clinical factors, the nomogram model had higher OS prediction accuracy at 1, 3, and 5 years (**Figures 6E-G**). This prognostic model with multiple clinical factors also had a greater net benefit in predicting the prognosis (**Figures 6H-J**). Based on the evidence above, the cuproptosis score and its constructed nomogram model can accurately predict the prognosis of bladder cancer patients and can be used as a clinical decision-making tool (*p*-value < 0.05 was considered statistically significant).

**Table 1:** Relationship between cuproptosis score and clinicopathological features of BLCA in TCGA.

Covariates	Total	High score	Low score	*p*-Value
		No. (%)	No. (%)	
**Age (years)**				0.3503
** <**65	150 (37.22%)	26 (32.5%)	124 (38.39%)	
** >**65	253 (62.78%)	54 (67.5%)	199 (61.61%)	
**Gender**				0.6687
Female	105 (26.05%)	19 (23.75%)	86 (26.63%)	
Male	298 (73.95%)	61 (76.25%)	237 (73.37%)	
**Status**				0.001
Alive	176 (43.67%)	50 (62.5%)	126 (39.01%)	
Dead	227 (56.33%)	30 (37.5%)	197 (60.99%)	
**Grade**				0.1354
Low grade	20 (4.96%)	1 (1.25%)	19 (5.88%)	
High grade	380 (94.29%)	78 (97.5%)	302 (93.5%)	
Unknown	3 (0.74%)	1 (1.25%)	2 (0.62%)	
**T classification**				0.3313
T0	1 (0.25%)	1 (1.25%)	0 (0%)	
T1	3 (0.74%)	1 (1.25%)	2 (0.62%)	
T2	117 (29.03%)	19 (23.75%)	98 (30.34%)	
T3	192 (47.64%)	41 (51.25%)	151 (46.75%)	
T4	57 (14.14%)	12 (15%)	45 (13.93%)	
Unknown	32 (7.94%)	6 (7.5%)	26 (8.05%)	
**N classification**				0.1159
N0	235 (58.31%)	47 (58.75%)	188 (58.2%)	
N1	44 (10.92%)	8 (10%)	36 (11.15%)	
N2	75 (18.61%)	20 (25%)	55 (17.03%)	
N3	7 (1.74%)	0 (0%)	7 (2.17%)	
NX	36 (8.93%)	3 (3.75%)	33 (10.22%)	
Unknown	6 (1.49%)	2 (2.5%)	4 (1.24%)	
**M classification**				0.02
M0	195 (48.39%)	49 (61.25%)	146 (45.2%)	
M1	11 (2.73%)	1 (1.25%)	10 (3.1%)	
MX	194 (48.14%)	28 (35%)	166 (51.39%)	
Unknown	3 (0.74%)	2 (2.5%)	1 (0.31%)	
**TNM stage**				0.3453
I	2 (0.5%)	1 (1.25%)	1 (0.31%)	
II	128 (31.76%)	20 (25%)	108 (33.44%)	
III	140 (34.74%)	30 (37.5%)	110 (34.06%)	
IV	131 (32.51%)	28 (35%)	103 (31.89%)	
Unknown	2 (0.5%)	1 (1.25%)	1 (0.31%)	

BLCA, bladder cancer; TCGA, The Cancer Genome Atlas.

### Cuproptosis score is correlated with tumor microenvironment characteristics and mutation of bladder cancer

**Figure 7:**
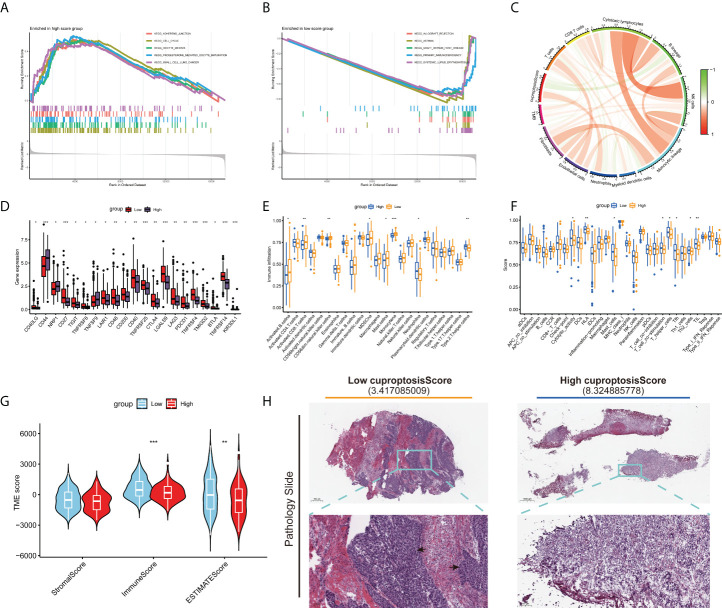
Distinct TME characteristics and mutation of BLCA patients according to the cuproptosis score. GSEA shows the different pathways significantly enriched in the **(A)** high and **(B)** low cuproptosis score groups. **(C)** Correlations between cuproptosis score and immune cells. **(D)** Expression of immune checkpoints in the high and low score groups. Boxplots show **(E)** abundance of 23 infiltrating immune cell types and **(F)** differences in immune scores in the two cuproptosis score groups. **(G)** Correlations between cuproptosis score and both immune and stromal scores. **(H)** Images representing the pathological H&E staining variations between the high and low cuproptosis score groups (TCGA database). (*p < 0.05; **p < 0.01; ***p < 0.001; Ns, not significant). TME, tumor microenvironment; BLCA, bladder cancer; GSEA, gene set enrichment analysis; TCGA, The Cancer Genome Atlas.

The results of GSEA suggested that the pathways associated with tumor growth and invasion were significantly enriched in the high cuproptosis score group **Figure 7A**), but immune-related pathways tended to be enriched in the low cuproptosis group (**Figure 7B**). Next, we examined the relationship between cuproptosis score and immune cells, which is shown in **Figure 7C**. Then, we examined the relationship between immune checkpoints and cuproptosis scores. In **Figure 7D**, there were 21 differentially expressed immune checkpoints in the two groups, including PD-1 and CTLA-4. **Figures 7E, F** indicate a correlation between low cuproptosis scores and higher levels of immune cell infiltration and immune function, consistent with previous GSEAs. In both TMEscore and histopathological sections, the group with low cuproptosis showed higher immune cell infiltration (**Figures 7G, H**). We also analyzed the survival of different subgroups of TMB to explore their impact on the prognosis of patients with BLCA. Compared to patients with high TMB, those with low TMB have a worse prognosis (**Figure S4A**). Afterward, the survival of patients with BLCA combined with TMB and cuproptosis score was analyzed, and the prognostic benefit in the low TMB group was counteracted by the cuproptosis score (**Figure S4B**). Moreover, we also investigated the distributional differences of somatic mutations between cuproptosis score groups in TCGA-BLCA samples. As shown in **Figures S4C, D**, the mutation rates of TP53, TTN, KMT2D, MUC16, and PIK3CA are higher than or equal to 20% in BLCA patients in both groups. Moreover, patients with a high cuproptosis score were more likely to have mutations in these genes than those with a low score (*p*-value < 0.05 was considered statistically significant).

### Predicting the sensitivity of bladder cancer patients to antitumor therapy with the cuproptosis score

**Figure 8:**
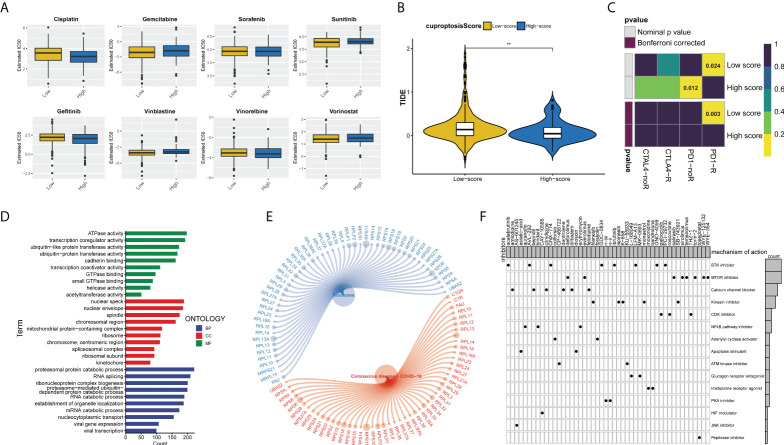
Cuproptosis score predicts the responsiveness of BLCA to chemotherapy, immunotherapy, and potential small-molecule compounds. **(A)** Relationships between cuproptosis score and chemotherapeutic sensitivity of BLCA. **(B)** Violin-plots show the differences in TIDE scores between the two cuproptosis score groups. **(C)** The submap algorithm predicts the probability of anti-PD1 and anti-CTLA4 immunotherapy response in high and low cuproptosis groups. The low cuproptosis score group may benefit more from PD-1 treatment (Bonferroni corrected *p* = 0.003). R, response. **(D)** GO enrichment analysis ranked by adjusted *p*-value and **(E)** KEGG pathway enrichment analysis results of DEGs between the two cuproptosis score groups. **(F)** Dots distribution heatmap depicting small-molecule compounds and their shared drug mechanisms of action (rows) through the CMap database. (**p < 0.01). BLCA, bladder cancer; GO, Gene Ontology; KEGG, Kyoto Encyclopedia of Genes and Genomes; DEGs, differentially expressed genes; CMap, Connectivity Map.

We assessed the half-maximal inhibitory concentration (IC50) values for eight different chemotherapeutic drugs, including cisplatin, gemcitabine, sunitinib, gefitinib, vinblastine, vinorelbine, and vorinostat, to predict the likelihood of BLCA patients responding to antitumor therapy with the cuproptosis score. The results showed that sunitinib (*p* = 0.019) and vinblastine (*p* = 0.01) had significantly higher IC50 estimates in the high cuproptosis score group than in the low cuproptosis score group, but no significant differences were seen in the other drugs (**Figure 8A**). Furthermore, ICB treatment efficacy was evaluated using the tumor immune dysfunction and exclusion (TIDE) algorithm between the two cuproptosis score clusters. BLCA patients with low cuproptosis scores had higher TIDE scores than those with high scores (*p* = 0.01, **Figure 8B**). In addition, it showed that the low cuproptosis score subtype may benefit more from anti-PD1 treatment (Bonferroni corrected *p* = 0.003, **Figure 8C**). In conclusion, the high cuproptosis score group may be more sensitive to chemotherapy, whereas the low cuproptosis score group is more sensitive to anti-PD1 immunotherapy. We further analyzed the molecular differences between the high and low cuproptosis subgroups in order to identify the small-molecule drugs that could be used to treat BLCA. Finally, 4,506 genes with *p* < 0.05 and |log2-fold change (FC)| ≥ 1 were determined between the two subgroups, including 1,861 upregulated genes and 2,645 downregulated genes (**Table S4**). GO enrichment analysis showed that DEGs were significantly enriched in energy metabolism, RNA splicing, and viral gene expression (**Figure 8D**). These DEGs were also significantly correlated with ribosomes and coronavirus disease 2019 (COVID-19) signaling pathways (**Figure 8E**). We uploaded the 150 upregulated and 150 downregulated genes to the Connectivity Map (CMap) database (https://portals.broadinstitute.org/cmap/) of small-molecule drugs and examined their mechanisms of action. There were 48 potential small-molecule drugs and 15 potential drug mechanisms identified (**Figure 8F**). There was a high correlation between BTK and mTOR pathway inhibitors, suggesting the huge potential value of these two pathways (*p*-value < 0.05 was considered statistically significant).

## Discussion

The latest research has identified a new form of cell death caused by copper in human cells, known as copper-induced cell death (cuproptosis) (8). The process of cuproptosis is closely related to mitochondrial respiration. The copper that is too abundant within cells can be transported to the mitochondria by ionophores and directly bind to lipoylated components of the tricarboxylic acid cycle, resulting in the accumulation of lipoylated proteins and loss of iron–sulfur cluster proteins, which leads to proteotoxic stress and ultimately to cell death. A growing body of evidence shows that unbalanced copper homeostasis can affect tumor growth and induce tumor cell death (20), and copper plays an indispensable role in tumor immunity and antitumor therapy (21, 22). In addition, the development of diverse biomaterials and nanotechnology that allow copper to be fabricated into different structures to achieve its therapeutic action is worthy of attention (20). However, its role in the development of TME and its potential therapeutic value in bladder cancer remain unclear. Here, we comprehensively described how cuproptosis patterns in BLCA are clinically significant and how they may be related to TME characteristics. In addition, the cuproptosis scoring system was proposed to evaluate individual cuproptosis to improve understanding of TME and assist doctors in developing more effective immunotherapeutic strategies.

We investigated transcriptional changes and CUG expression using TCGA-BLCA cohort in this study. In line with previous studies (3), most of the CUGs were altered, including CDKN2A. By using an unsupervised clustering approach, we then developed two BLCA molecular patterns according to CUGs’ mRNA expression profiles. The prognosis, immune infiltration, and immune function of the two groups were significantly different. The response to immunotherapy may be heavily influenced by genomic alterations in BLCA. Based on DEGs associated with cluster signature, four gene clusters with differential clinical outcome, immune activity, and immune function were identified for BLCA. The cuproptosis score was established through the Boruta algorithm. In clinical application, transcriptome sequencing can be performed on pathological samples from bladder cancer patients to determine the cuproptosis scores of patients. A high cuproptosis score was associated with a shorter overall survival time, indicating that a high cuproptosis score may predict poor outcomes. As a result of the GSEA enrichment analysis, cancer and immune-related pathways were significantly enriched, suggesting that cuproptosis is involved in tumor development and TME.

Similarly, cuproptosis scores correlated significantly with BLCA clinicopathological features. We found that the cuproptosis score is an independent predictor of survival outcomes among BLCA patients after controlling for confounders. The receiver operating characteristic (ROC) curves confirmed its favorable predictive validity for 1-, 3-, and 5-year OS. In light of these data, the cuproptosis score may have robust predictive power for the prognosis of BLCA patients. Genetic mutations that caused cancer are significantly associated with copper (23). We found a significant genetic mutation difference between groups with high and low cuproptosis scores in our study. BLCA patients with high TMB have been shown to have better survival outcomes (24), which is consistent with our results. There was a significant survival advantage for patients with a lower cuproptosis score compared to those with a lower TMB score, suggesting that the cuproptosis score could predict immunotherapy response independently of TMB.

The immune response plays a leading role in tumorigenesis and can often be used as a target for tumor therapy. In the TME, immune cells and stromal cells are identified as major components (25, 26). Immune cell infiltration was associated with bladder cancer survival, and high CD8+ T-cell infiltration predicted a favorable prognosis, confirming our analysis (27, 28). This suggests that cuproptosis may be involved in the regulation of TME, especially CD8+ T cells, and therefore contribute to tumor growth and progression. In previous studies, the reactivation of CD8+ T cells predicted the efficacy of immunotherapy. Consequently, targeting cuproptosis may be an effective and novel therapeutic strategy for the treatment of BLCA.

Chemotherapy, immunotherapy, and targeted therapy can limit tumor progression and improve prognosis in patients with advanced bladder cancer (29). At present, BLCA’s decreasing sensitivity to chemotherapy is causing widespread concern (30). Patients with different cuproptosis score groups were identified as potentially sensitive to these agents, and the combination of these agents with cuproptosis-targeted drugs may help mitigate resistance mechanisms and improve clinical outcomes. In addition, immunotherapy requires specific predictive models to be effective. The TIDE method has been used to predict BLCA patients’ ICB response. In this study, we observed that BLCA patients with a low cuproptosis score had a higher TIDE score and a stronger positive response to anti-PD1 therapy than those with a high cuproptosis score. The low-score group consistently had higher levels of T-cell infiltration. It implies that the cuproptosis score could be a screening tool for ICB patients.

Subsequently, 1,861 upregulated genes and 2,645 downregulated genes were identified in the low and high cuproptosis score groups, respectively. The results of enrichment analysis showed that these specific DEGs were enriched in energy metabolism, RNA splicing, and viral gene expression. Additionally, KEGG enrichment analysis revealed that the DEGs were significantly enriched in ribosomes and the COVID-19 signaling pathway. The above results suggest that cuproptosis may be involved in more than tumor development, including the occurrence and development of viral diseases, as documented by relevant reports (31). Despite increasing support for targeted therapies in cancer, cuproptosis-targeted drugs are scarce at present. In our research, we identified 48 genetic and pharmacological inhibitors targeting cuproptosis, including BTK and mTOR inhibitors, covering 15 mechanisms of action. The results of this study provide valuable evidence for targeted treatment of BLCA.

There are still some deficiencies in the study. First of all, our analysis was a secondary analysis of the data from public databases. These retrospective data were subject to selection bias, which affected the accuracy of the analysis results. Hence, well-designed prospective studies are needed to validate our findings. In addition, to fully comprehend the clinical significance of cuproptosis, more clinical factors should be analyzed. Unfortunately, the collection of these variables in public databases is incomplete.

## Conclusions

To sum up, we systematically analyzed the effects of cuproptosis on BLCA and provided a clear explanation of the broad regulatory mechanisms of cuproptosis in the TME, clinicopathological features, and prognosis. In addition, we demonstrated the potency of CUGs as biomarkers of therapeutic response. Consequently, an integrated evaluation of cuproptosis scores of each BLCA patient is of great clinical importance and can be used to develop personalized immunotherapy strategies for these patients.

## Data availability statement

Publicly available datasets were analyzed in this study. This data can be found here: the TCGA-BLCA (https://portal.gdc.cancer.gov/) and GSE16671 and GSE13507 in GEO (https://www.ncbi.nlm.nih.gov/geo/).

## Author contributions

QS: data curation, software, and writing—original draft preparation. RZ: visualization, software, and formal analysis. FS: writing—review and editing, and project administration. WF: conceptualization, methodology, writing—reviewing and editing, and funding acquisition. All authors contributed to the article and approved the submitted version.

## Funding

This work was supported by the Basic and Applied Basic Research Fund of Guangdong Province (grant number 2021A1515010860) and Guangzhou Municipal Science and Technology Project (grant numbers 202102010170 and 202102010236).

## Acknowledgments

We acknowledge and appreciate the help of FigureYa in plotting the figures. We thank Bullet Edits Limited for the linguistic editing and proofreading of the manuscript.

## Conflict of interest

The authors declare that the research was conducted in the absence of any commercial or financial relationships that could be construed as a potential conflict of interest.

## Publisher’s note

All claims expressed in this article are solely those of the authors and do not necessarily represent those of their affiliated organizations, or those of the publisher, the editors and the reviewers. Any product that may be evaluated in this article, or claim that may be made by its manufacturer, is not guaranteed or endorsed by the publisher.

## Supplementary material

Supplementary Figure 1 The prognostic value of 10 CUGs in patients with BLCA based on the TCGA-BLCA cohort and GSE13507 dataset.

Supplementary Figure 2
**(A, B)** GO and KEGG enrichment analyses of DEGs among two cuproptosis subtypes.

Supplementary Figure 3 Consensus clustering analysis for BLCA samples from TCGA database based on prognostic DEGs between the two cuproptosis subtypes. **(A)** Cumulative distribution function (CDF) when different k values. **(B)** Delta area plot shows relative change in area under CDF curve when at a certain k value compared with k-1. **(C)** Differences in the expression of 10 CUGs among the four gene clusters. (p < 0.05 *; p < 0.01 **; p < 0.001 ***)

Supplementary Figure 4 TMB and genetic mutations of BLCA according to the two cuproptosis groups. **(A)** Kaplan-Meier analysis of the OS between the low- and high-TMB groups in BLCA samples. **(B)** Kaplan-Meier analysis among four patient groups stratified by both TMB and cuproptosis score. **(C, D)** The waterfall plot of somatic mutation features established with low and high cuproptosis score groups.
