# Supplementary figures and images for: Cuproptosis scoring system to predict the clinical outcome and immune response in bladder cancer
Source: Front Immunol. 2022 Aug 4;13:958368. doi: 10.3389/fimmu.2022.958368 (PMC9386055; doi:10.3389/fimmu.2022.958368)

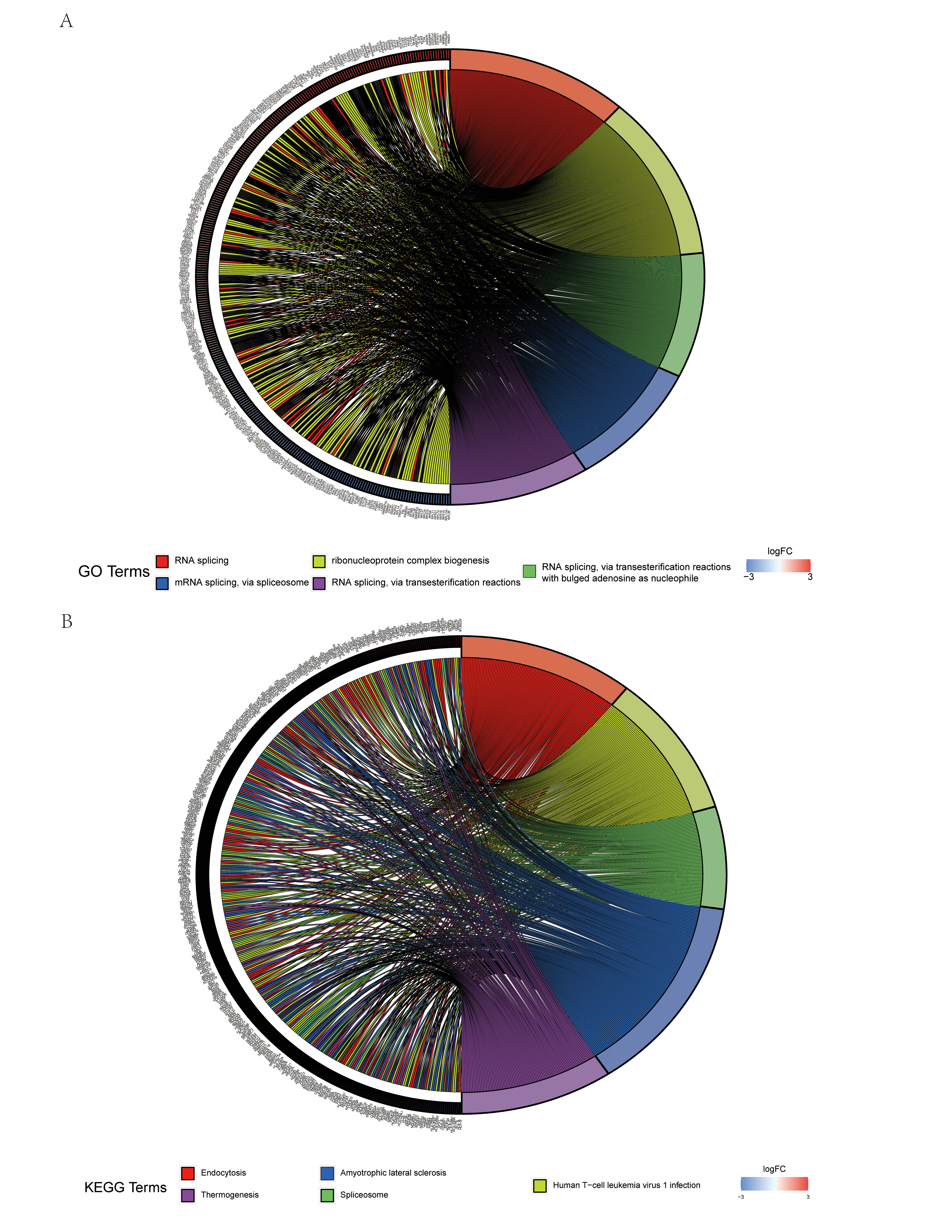

Supplement: Supplementary Figure 2 — (A, B) GO and KEGG enrichment analyses of DEGs among two cuproptosis subtypes. [file Image_2.jpeg]

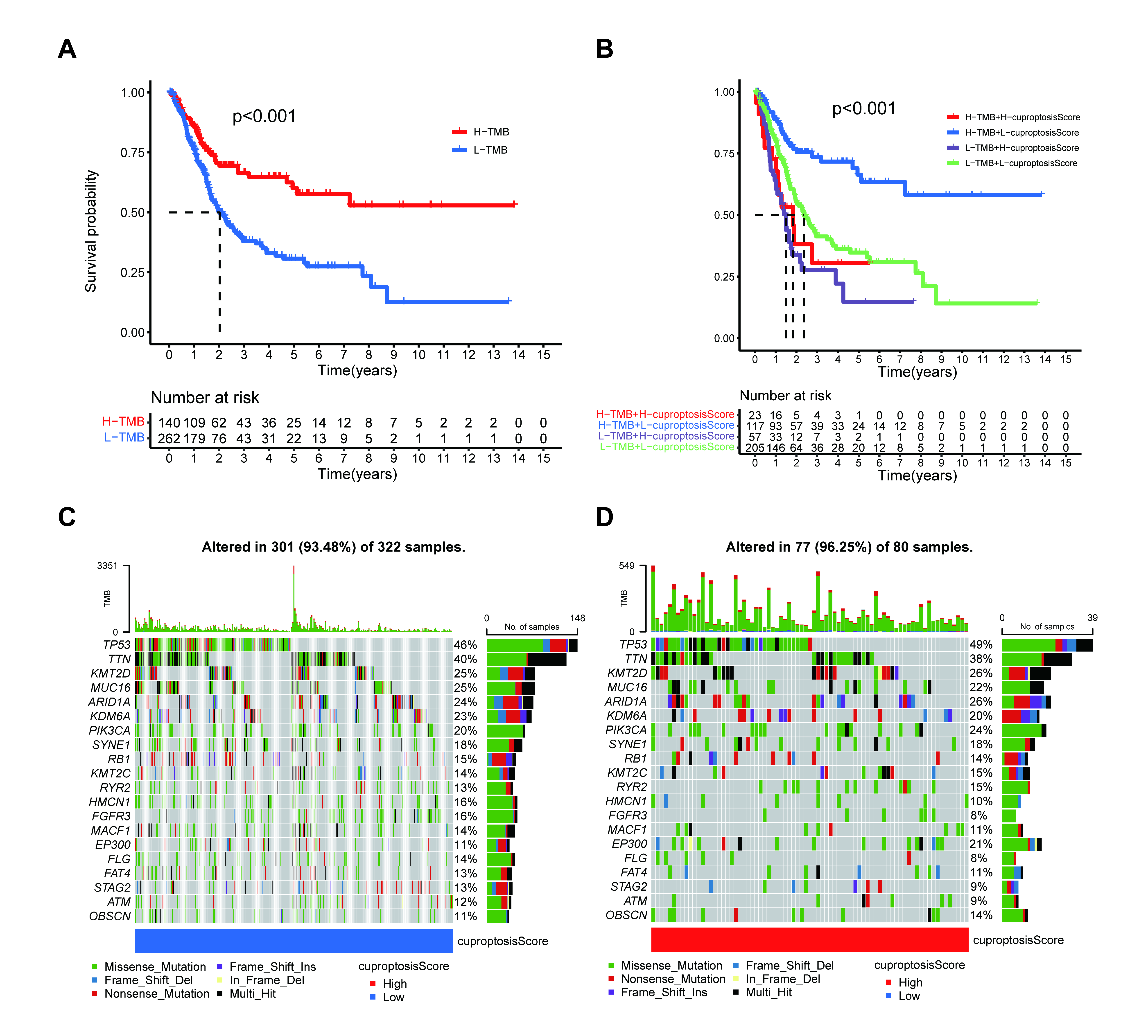

Supplement: Supplementary Figure 4 — TMB and genetic mutations of BLCA according to the two cuproptosis groups. (A) Kaplan-Meier analysis of the OS between the low- and high-TMB groups in BLCA samples. (B) Kaplan-Meier analysis among four patient groups stratified by both TMB and cuproptosis score. (C, D) The waterfall plot of somatic mutation features established with low and high cuproptosis score groups. [file Image_4.tif]
